# In Vitro Metabolism of 25B-NBF, 2-(4-Bromo-2,5-Dimethoxyphenyl)-*N*-(2-Fluorobenzyl)ethanamine, in Human Hepatocytes Using Liquid Chromatography–Mass Spectrometry

**DOI:** 10.3390/molecules24040818

**Published:** 2019-02-25

**Authors:** Ju-Hyun Kim, Sunjoo Kim, Jaesin Lee, Sangwhan In, Yong-Yeon Cho, Han Chang Kang, Joo Young Lee, Hye Suk Lee

**Affiliations:** 1 BK21 PLUS Team for Creative Leader Program for Pharmacomics-based Future Pharmacy, College of Pharmacy, The Catholic University of Korea, Bucheon 14662, Korea; jhkim@yu.ac.kr (J.-H.K.); sjkim712@catholic.ac.kr (S.K.); yongyeon@catholic.ac.kr (Y.-Y.C.); hckang@catholic.ac.kr (H.C.K.); joolee@catholic.ac.kr (J.Y.L.); 2 College of Pharmacy, Yeungnam University, Gyeongsan 38541, Korea; 3 National Forensic Service, Wonju 24460, Korea; ljstrust@korea.kr (J.L.); swin@korea.kr (S.I.)

**Keywords:** 25B-NBF metabolism, liquid chromatography-high resolution mass spectrometry, human hepatocyte, CYP, UGT

## Abstract

25B-NBF, 2-(4-bromo-2,5-dimethoxyphenyl)-*N*-(2-fluorobenzyl)ethanamine, is a new psychoactive substance classified as a phenethylamine. It is a potent agonist of the 5-hydroxytryptamine receptor, but little is known about its metabolism and elimination properties since it was discovered. To aid 25B-NBF abuse screening, the metabolic characteristics of 25B-NBF were investigated in human hepatocytes and human cDNA-expressed cytochrome P450 (CYP) and UDP-glucuronosyltransferase (UGT) enzymes using liquid chromatography–high resolution mass spectrometry. At a hepatic extraction ratio of 0.80, 25B-NBF was extensively metabolized into 33 metabolites via hydroxylation, *O*-demethylation, bis-*O*-demethylation, *N*-debenzylation, glucuronidation, sulfation, and acetylation after incubation with pooled human hepatocytes. The metabolism of 25B-NBF was catalyzed by CYP1A1, CYP1A2, CYP2B6, CYP2C9, CYP2C19, CYP2D6, CYP2J2, CYP3A4, and UGT2B7 enzymes. Based on these results, it is necessary to develop a bioanalytical method for the determination of not only 25B-NBF but also its metabolites in biological samples for the screening of 25B-NBF abuse.

## 1. Introduction

New psychoactive substances (NPSs) are abused compounds with effects similar to those of controlled drugs such as cannabis, morphine, cocaine, and amphetamine-type stimulants. Since the United Nations Office on Drugs and Crime (UNODC) launched the international NPS monitoring system in 2009, the amount of NPSs has increased by 3.7 times to 479 substances in 2017 [1]. Likewise, the European Monitoring Centre for Drugs and Drug Addiction (EMCDDA) was monitoring over 620 NPS at the end of 2016 and stated that more than 70% of these had been newly detected in the previous 5 years [2]. NPSs can be classified based on their chemical structure as synthetic cannabinoids, synthetic cathinones, tryptamines, phenethylamines, and others. Although synthetic cannabinoids remain the most commonly abused group, recently phenethylamine abuse has been on the rise, accounting for 28.4% of cases of NPS abuse at the end of 2017 according to the UNODC [1].

Phenethylamines are typical agonists of the 5-hydroxytryptamine 2 (5-HT2) receptor and their structure–activity relationships (SARs) are well-characterized [3]. Because *N*-benzyl substitution of phenethylamine results in a significant increase in binding affinity to 5-HT2A receptor and receptor activities, several *N*-benzyl-related analogs with methoxy, hydroxy, or fluorine moieties have been synthesized and their SARs have been evaluated [4,5]. 25B-NBF, 2-(4-bromo-2,5-dimethoxyphenyl)-*N*-(2-fluorobenzyl)ethanamine, is an *N*-fluorobenzyl derivative of 2C-B [2-(4-bromo-2,5-dimethoxyphenyl)ethanamine] that binds to human 5-HT2A receptors and rat 5-HT2C receptors with pK_i_ values of 8.57 and 7.77, respectively [4]. 25B-NBF is intended for research and forensic applications but is classified as a controlled substance in Sweden, the United Kingdom, and the Republic of Korea because of its potential for abuse [6,7]. Also, severe intoxication cases and the in vitro and in vivo metabolism of a structurally related substance, 25B-NBOMe [2-(4-bromo-2,5-dimethoxyphenyl)-*N*-(2-methoxybenzyl)-ethanamine], have been reported [8,9,10,11,12,13,14,15], but there are no published reports on the metabolism of 25B-NBF in humans and experimental animals.

For the control of illegal substances, it is necessary to develop bioanalytical methods for the sensitive and selective detection of illegal substances. Because many illegal substances are extensively metabolized, their metabolic profiles have been characterized to develop the bioanalytical methods for the simultaneous determination of illegal drugs and metabolites in forensic and emergency cases [13,14,15,16,17,18]. Therefore, it is necessary to understand the metabolic pathway of 25B-NBF in order to develop a bioanalytical method for monitoring 25B-NBF abuse. The purposes of this study were to identify in vitro metabolic profiles of 25B-NBF using human hepatocytes, the gold standard in vitro metabolism model, and liquid chromatography–high resolution mass spectrometry (LC–HRMS) [17,18,19] and to characterize the specific cytochrome P450 (CYP) and uridine 5′-diphospho-glucuronosyltransferase (UGT) enzymes responsible for 25B-NBF metabolism using human cDNA-expressed CYPs and UGTs in order to predict the pharmacokinetics and drug-interaction potential of 25B-NBF.

## 2. Results

### 2.1. Metabolic Stability of 25B-NBF and Prediction of Its Hepatic Clearance

**Figure 1:**
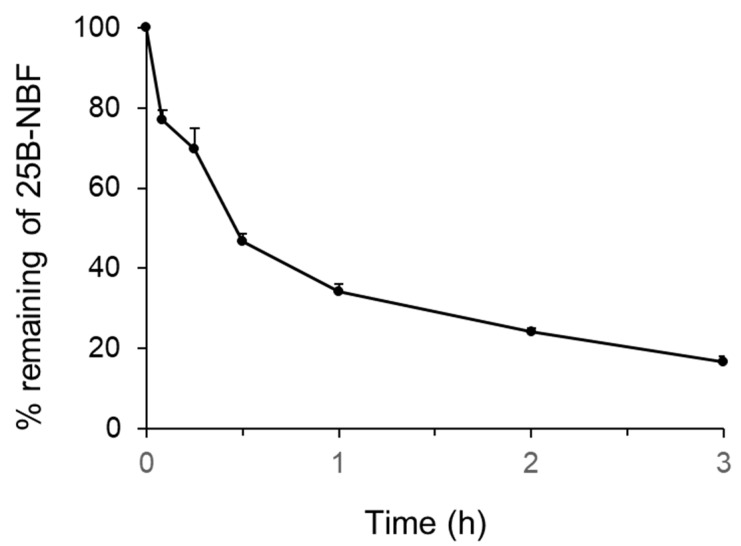
Percentage of 25B-NBF [2-(4-bromo-2,5-dimethoxyphenyl)-*N*-(2-fluorobenzyl)ethanamine] remaining after incubation with human hepatocytes at 37 °C in a CO_2_ incubator. The symbol and error bar represent mean and standard deviation, respectively (*n* = 3).

The metabolic stability of 25B-NBF in human hepatocytes is illustrated in Figure 1. After 3 h incubation, 16.6% of 25B-NBF remained in the human hepatocyte suspension. The elimination slope (*k*) of degradation was estimated by linear regression and the elimination half-life (t_1/2_) of 25B-NBF was 29.7 min. Intrinsic clearance (Cl_int_) and hepatic clearance (Cl_hep_) of 25B-NBF were estimated using the well-stirred model as 83.4 and 16.6 mL/min/kg, respectively. The hepatic extraction ratio of 25B-NBF was 0.80, indicating that it is extensively metabolized in the liver. The Cl_int_, Cl_hep_, hepatic extraction ratio of honokiol, a positive control, using human hepatocytes were 168.4 mL/min/kg, 18.4 mL/min/kg, and 0.89, respectively, comparable to a previous report [19]. 

### 2.2. Metabolite Identification of 25B-NBF

**Figure 2:**
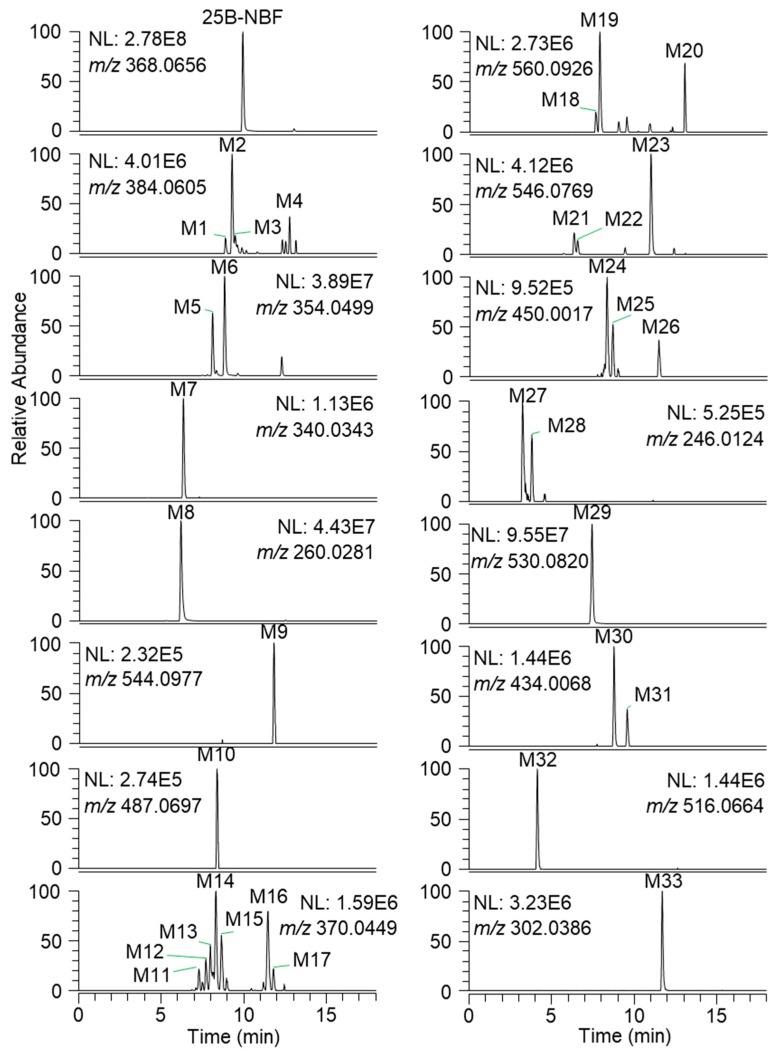
Representative extracted ion chromatograms of 25B-NBF and 33 metabolites identified after incubation with human hepatocytes for 2 h at 37 °C in a CO_2_ incubator.

The incubation of 25B-NBF with human hepatocytes for 2 h at 37 °C resulted in the formation of 33 metabolites from 25B-NBF. Representative extracted ion chromatograms of 25B-NBF and its metabolites are presented in Figure 2, and the retention times, molecular formulae, exact molecular ions ([M + H]^+^), mass accuracies, fragment ions, and biotransformation are summarized in Table 1.

**Figure 3:**
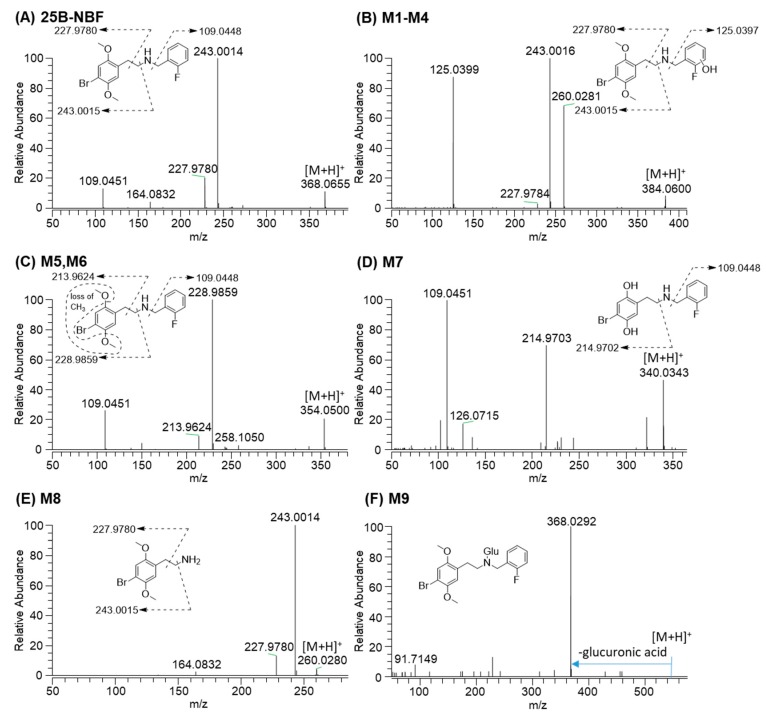
MS/MS spectra of 25B-NBF and typical metabolites identified after incubation with human hepatocytes for 2 h at 37 °C in a CO_2_ incubator. (**A**) 25B-NBF; (**B**) hydroxyl-25B-NBF (M1–M4); (**C**) *O*-demethyl-25B-NBF (M5 and M6); (**D**) bis-*O*-demethyl-25B-NBF (M7); (**E**) 2C-B (M8); (**F**) 25B-NBF glucuronide (M9).

25B-NBF produced an [M + H]^+^ ion at *m*/*z* 368.0656 and the isotope ion at *m*/*z* 370.0635 with similar intensity to the [M + H]^+^ ion owing to the characteristic isotope pattern of bromine. 25B-NBF produced three characteristic fragment ions at *m*/*z* 243.0015 [2-(4-bromo-2,5-dimethoxyphenyl)ethan-1-ylium], *m*/*z* 227.9780 [4-bromo-2,5-dimethoxyphenyl) methylium], and *m*/*z* 109.0448 [(2-fluorophenyl)methylium], which served as a marker of fragment ions for metabolite identification (Figure 3A).

M1–M4 produced an [M + H]^+^ ion at *m*/*z* 384.0605, 15.9949 amu higher than that of 25B-NBF, indicating that they were hydroxy-25B-NBF. M1–M4 produced product ions at *m*/*z* 260.0280, *m*/*z* 243.0015, *m*/*z* 227.9780, and *m*/*z* 125.0399 [(hydroxy-2-fluorophenyl)methylium], and therefore, M1–M4 were formed via hydroxylation at the fluorobenzyl moiety but the accurate hydroxylation position of each metabolite was not determined (Figure 3B).

M5 and M6 showed an [M + H]^+^ ion at *m*/*z* 354.0499, 14.0157 amu lower than 25B-NBF, indicating that they were *O*-demethyl-25B-NBF. Based on the product ions at *m*/*z* 228.9859 and *m*/*z* 213.9624, which were 14 amu lower than *m*/*z* 243.0015 and *m*/*z* 227.9780 ions of 25B-NBF, respectively, M5 and M6 appeared to be *O*-demethyl-25B-NBF (Figure 3C). However, the accurate demethylation position of M5 and M6 could not be determined because of the absence of authentic standards.

The [M + H]^+^ ion of M7 was observed at *m*/*z* 340.0343, 28.0313 amu lower than 25B-NBF, indicating bis-*O*-demethyl-25B-NBF, which was supported by the characteristic product ions at *m*/*z* 214.9703 [2-(4-bromo-2,5-dihydroxyphenyl)ethan-1-ylium] and *m*/*z* 109.0451 (Figure 3D).

M8 was identified as 2C-B [2-(4-bromo-2,5-dimethoxyphenyl)ethanamine] on the basis of its [M + H]^+^ ion at *m*/*z* 260.0281 and product ions at *m*/*z* 243.0014 (loss of NH_3_ from the [M + H]^+^ ion) and *m*/*z* 227.9780 (Figure 3E).

M9 produced an [M + H]^+^ ion at *m*/*z* 544.0977, 176.0321 amu higher than 25B-NBF, and a product ion at *m*/*z* 368.0292 (25B-NBF ion due to loss of glucuronic acid), suggesting that it was 25B-NBF *N*-glucuronide (Figure 3F).

M10 produced an [M + H]^+^ ion at *m*/*z* 487.0697, 119.0041 amu higher than 25B-NBF, and a product ion at *m*/*z* 243.0014 and *m*/*z* 227.9780, suggesting that M10 was a 25B-NBF cysteine conjugate. However, the accurate cysteine conjugation position was not identified (Figure S1A).

M11–M17 showed an [M + H]^+^ ion at *m*/*z* 370.0449, 1.9793 amu higher than 25B-NBF, suggesting that they were products of monohydroxylation and *O*-demethylation. Six metabolites (M11–M15, M17) produced the characteristic product ions at *m*/*z* 245.0046 (loss of fluorobenzyl moiety from [M + H]^+^ ion), *m*/*z* 228.9859, *m*/*z* 142.0663 (loss of 2-(4-bromo-hydroxymethoxyphenyl)ethane from [M + H]^+^ ion), and *m*/*z* 125.0398 (Figure S1B), indicating that M11–M15, and M17 were formed by *O*-demethylation at the dimethoxyphenyl moiety and hydroxylation at the fluorobenzyl moiety of 25B-NBF. M16 produced product ions at *m*/*z* 228.9859, *m*/*z* 214.9702, and *m*/*z* 109.0451 (Figure S1C), indicating that M16 was formed by *O*-demethylation and hydroxylation at the amino group of 25B-NBF. The exact positions of hydroxylation and *O*-demethylation for M11–M17 could not be identified.

M18–M20 produced an [M + H]^+^ ion at *m*/*z* 560.0926, 192.0270 amu higher than 25B-NBF, and product ions at *m*/*z* 384.0605 (loss of glucuronic acid from [M + H]^+^ ion), *m*/*z* 243.0015, and *m*/*z* 125.0399, suggesting that M18-M20 may be hydroxy-25B-NBF glucuronide via monohydroxylation at the fluorobenzyl moiety and glucuronidation (Figure S2A). The exact location of hydroxylation and glucuronidation could not be determined. 

M21–M23 produced [M + H]^+^ ion at *m*/*z* 546.0769 and the product ions at *m*/*z* 370.0449 (loss of glucuronyl moiety from the [M + H]^+^ ion), suggesting that they were hydroxy-*O*-demethyl-25B-NBF glucuronides. M21 and M23 also produced the product ions at *m*/*z* 352.0345 (loss of H_2_O from *m*/*z* 370.0449), *m*/*z* 228.9859, *m*/*z* 214.9702, and *m*/*z* 109.0448, suggesting glucuronidation of M16 (Figure S2B). M22 produced product ions at *m*/*z* 422.0442 (loss of hydroxyflourobenzyl moiety from the [M + H]^+^ ion), *m*/*z* 246.0124 (loss of glucuronosyl moiety from *m*/*z* 422.0442), *m*/*z* 228.9861, and *m*/*z* 125.0399 (Figure S2C).

M24–M26 produced an [M + H]^+^ ion at *m*/*z* 450.0017, 79.9568 amu higher than the [M + H]^+^ ion of hydroxy-*O*-demethyl-25B-NBF, and the product ions at *m*/*z* 370.0449 and *m*/*z* 228.9861, suggesting sulfation of hydroxy-*O*-demethyl-25B-NBF. M24 and M25 produced the product ion at *m*/*z* 125.0399 (Figure S3A), whereas M26 showed a *m*/*z* 109.0451 ion (Figure S3B), indicating that the positions of hydroxylation in M24 and M25 were different from those of M26.

M27 and M28 produced an [M + H]^+^ ion at *m*/*z* 246.0124, 14.0157 amu lower than M8, indicating *O*-demethyl-2C-B, and these metabolites were confirmed by the presence of a product ion at *m*/*z* 228.9858 (loss of NH_3_) and *m*/*z* 213.9597 (Figure S3C).

M29 produced an [M + H]^+^ ion at *m*/*z* 530.0820 and product ions at *m*/*z* 354.0499 (loss of glucuronyl moiety from [M + H]^+^ ion) and *m*/*z* 228.9859, indicating that M29 was *O*-demethyl-25B-NBF glucuronide (Figure S4A).

M30 and M31 produced an [M + H]^+^ ion at *m*/*z* 434.0068 and the product ions at *m*/*z* 354.0499 (loss of sulfate from the [M + H]^+^ ion), *m*/*z* 308.9427 [loss of (2-fluorophenyl)methanamine from the [M + H]^+^ ion), *m*/*z* 228.9859 (loss of sulfate from *m*/*z* 308.9427), and *m*/*z* 109.0451 (Figure S4B), indicating that M30 and M31 were *O*-demethyl-25B-NBF sulfates formed by sulfation at the OH group.

M32 produced an [M + H]^+^ ion at *m*/*z* 516.0664 and product ions at *m*/*z* 340.0341 (loss of glucuronyl moiety from the [M + H]^+^ ion), *m*/*z* 214.9701, and *m*/*z* 109.0451, indicating that M32 was bis-*O*-demethyl-25B-NBF (M7) glucuronide (Figure S4C).

M33 produced an [M + H]^+^ ion at *m*/*z* 302.0386 and product ions at *m*/*z* 260.0280 (loss of acetyl moiety from the [M + H]^+^ ion), *m*/*z* 243.0014 (loss of NH_3_ from the [M + H]^+^ ion) and *m*/*z* 227.9781, indicating that M33 was acetyl-2C-B (Figure S4D). 

### 2.3. Screening of CYPs and UGTs Responsible for the Metabolism of 25B-NBF

**Figure 4:**
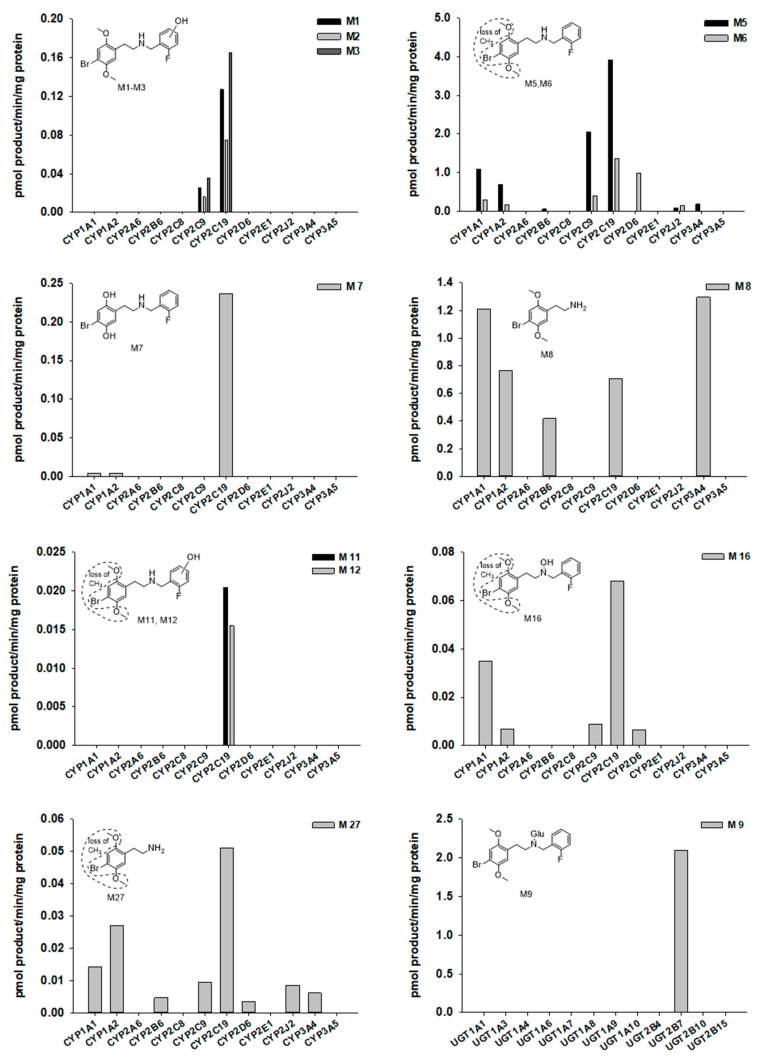
Formation rates of several 25B-NBF metabolites obtained from incubation of 25B-NBF with human cDNA-expressed CYPs 1A1, 1A2, 2A6, 2B6, 2C8, 2C9, 2C19, 2D6, 2E1, 2J2, 3A4, or 3A5, and UGTs 1A1, 1A3, 1A4, 1A6, 1A7, 1A8, 1A9, 1A10, 2B4, 2B7, 2B10, or 2B15. No data means that metabolite is not detected in recombinant CYPs or UGTs.

To characterize CYP enzymes involved in 25B-NBF metabolism, 25B-NBF (10 µM) was incubated with major human cDNA-expressed CYP1A1, CYP1A2, CYP2A6, CYP2B6, CYP2C8, CYP2C9, CYP2C19, CYP2D6, CYP2E1, CYP2J2, CYP3A4, or CYP3A5 in the presence of reduced form of nicotinamide adenine dinucleotide phosphate (NADPH). The metabolism of 25B-NBF was mediated by CYP1A1, CYP1A2, CYP2B6, CYP2C9, CYP2C19, CYP2D6, CYP2J2, and CYP3A4 (Figure 4). Some metabolites such as M4, M9, M10, M13, M14, M15, and M17 were not detected after incubation of 25B-NBF with recombinant CYP enzyme incubates.

UGT enzymes responsible for the glucuronidation of 25B-NBF were investigated with incubation of 25B-NBF in major human cDNA-expressed UGT1A1, UGT1A3, UGT1A4, UGT1A6, UGT1A7, UGT1A8, UGT1A9, UGT1A10, UGT2B4, UGT2B7, UGT2B10, and UGT2B15 in the presence of UDPGA. The formation of 25B-NBF glucuronide (M9) was solely mediated by UGT2B7 (Figure 4). However, other glucuronides of phase I metabolites of 25B-NBF could not be identified using recombinant UGT enzymes because of no availability of authentic metabolite standards.

## 3. Discussion

The in vitro metabolism of 25B-NBF, including metabolic stability and metabolite identification, was investigated using human hepatocytes. 25B-NBF was extensively metabolized in human hepatocytes, resulting in an elimination half-life and hepatic extraction ratio of 29.7 min and 0.80, respectively. The structure-related derivatives such as 25B-NBOMe, 25C-NBOMe, and 25I-NBOMe have been reported to undergo extensive metabolism in human, mouse, and rat [13,14,15,20].

**Figure 5:**
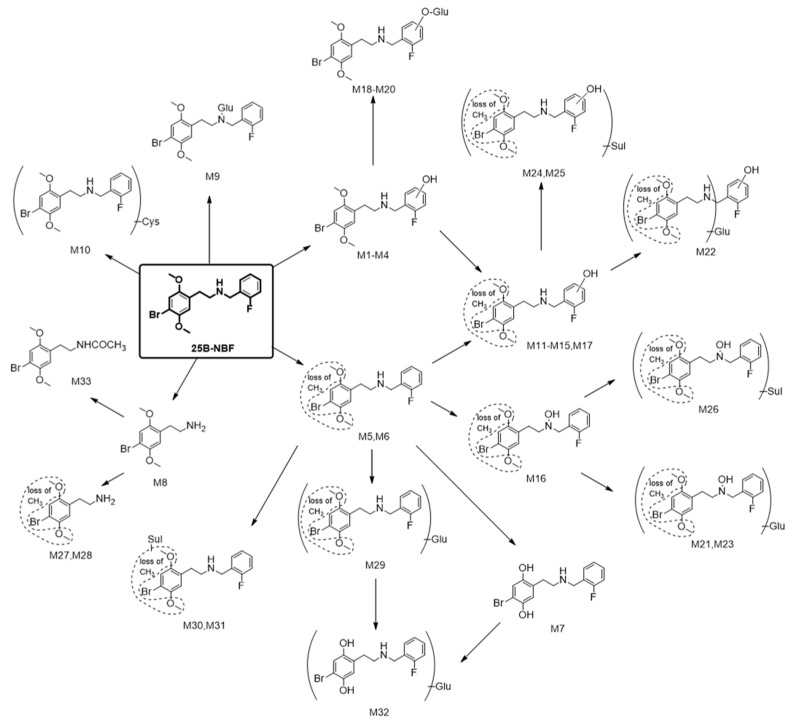
Proposed metabolic pathways of 25B-NBF in human hepatocytes. Glu: Glucuronide; Sul: Sulfate; Cys: Cysteine.

In terms of metabolite identification, the bromine atom of 25B-NBF is important for MS spectral interpretation due to the characteristic 1:1 isotopic ratio of ^79^Br and ^81^Br. 25B-NBF was metabolized into 33 metabolites by human hepatocytes: four hydroxy-25B-NBF (M1–M4), two *O*-demethyl-25B-NBF (M5 and M6), bis-*O*-demethyl-25B-NBF (M7), 2C-B (M8), 25B-NBF glucuronide (M9), 25B-NBF cysteine conjugate (M10), seven hydroxy-*O*-demethyl-25B-NBF (M11–M17), three hydroxy-25B-NBF glucuronides (M18–M20), three hydroxy-*O*-demethyl-25B-NBF glucuronides (M21–M23), three hydroxy-*O*-demethyl-25B-NBF sulfates (M24–M26), two *O*-demethyl-M8 (M27 and M28), *O*-demethyl-25B-NBF glucuronide (M29), two *O*-demethyl-25B-NBF sulfates (M30 and M31), M7 glucuronide (M32), and acetyl-M8 (M33) (Figure 5).

The metabolic pathways of 25B-NBF were hydroxylation, demethylation, *N*-dearylation, glucuronidation, sulfation, cysteine conjugation, and acetylation alone or in combination, comparable to those of structurally related derivatives such as 25B-NBOMe, 25C-NBOMe, 25I-NBOMe, and 25I-NBOH [13,14,15,20,21,22].

Multiple CYPs including CYP1A1, CYP1A2, CYP2B6, CYP2C9, CYP2C19, CYP2D6, CYP2J2, and CYP3A4 were responsible for the metabolism of 25B-NBF (Figure 4). The hydroxylation of the fluorobezyl moiety of 25B-NBF to M1–M3 was catalyzed by CYP2C9 and CYP2C19. CYP2C19 also played a major role in the demethylation of 25B-NBF to M5, M6, and M7 with minor contribution of CYP1A1, CYP1A2, CYP2C9, CYP2D6, CYP2J2, and CYP3A4. *N*-dearylation to M8 was mediated by several CYPs including CYP1A1, CYP1A2, CYP2B6, CYP2C19, and CYP3A4. The glucuronidation of 25B-NBF to 25B-NBF glucuronide (M9) was catalyzed by UGT2B7.

*N*-dearylation of 25B-NBF to 2C-B (M8), which is a psychedelic phenethylamine that is highly abused worldwide [23,24], was catalyzed by multiple CYPs, including CYP1A1/2, CYP2B6, CYP2C9, and CYP3A4. In previous reports, 2C-B was extensively metabolized into eight metabolites via *O*-demethylation, oxidative deamination, and amine oxidation catalyzed by monoamine oxidase, aldehyde dehydrogenase, alcohol dehydrogenase, and CYP enzymes [25,26,27,28]. In the present study, 2C-B (M8) and its three metabolites including *O*-demethyl-2C-B (M27 and M28) and an acetyl-2C-B (M33) were identified as the metabolites of 25B-NBF. These results suggest that abuse of 2C-B or 25B-NBF can be detected by 2C-B and a few other metabolites that persist in forensic biological samples.

## 4. Materials and Methods

### 4.1. Materials

25B-NBF was obtained from the Cayman Chemical Company (Ann Arbor, MI, USA). LiverPool™ cryopreserved human hepatocytes (50-donor pooled, lot no. HQE), InVitroGRO™ HT Medium, and InVitroGRO™ KHB were purchased from BioIVT (Brussels, Belgium). Honokiol (positive control in metabolic stability assays), ketoconazole (internal standard (IS) for metabolic stability), nicotinamide adenine dinucleotide phosphate, NADPH, glucose-6-phosphate, glucose-6-phosphate dehydrogenase, uridine 5′-diphosphoglucuronic acid (UDPGA), and ProteoMass™ LTQ/FT-Hybrid ESI Pos. Mode CalMix were purchased from Sigma-Aldrich (St. Louis, MO, USA). Homoegonol (IS for metabolic enzyme characterization) was purchased from Toronto Research Chemicals Inc. (Toronto, ON, Canada). LC–MS grade acetonitrile, methanol, and water were obtained from Fisher Scientific (Fair Lawn, NJ, USA). Human cDNA-overexpressed CYP isozymes (1A1, 1A2, 2A6, 2B6, 2C8, 2C9, 2C19, 2D6, 2E1, 2J2, 3A4, and 3A5), and human cDNA-overexpressed UGT isozymes (1A1, 1A3, 1A4, 1A6, 1A7, 1A8, 1A9, 1A10, 2B4, 2B7, 2B10, and 2B15) were obtained from Corning Life Sciences (Woburn, MA, USA). Other chemicals used were of the highest grade available. 

### 4.2. Metabolic Stability of 25B-NBF in Human Hepatocytes

Pooled cryopreserved human hepatocytes were carefully thawed in thawing medium and resuspended in Krebs–Henseleit buffer to a final density of 1.0 × 10^6^ cells/mL. Then a 60 μL aliquot of this hepatocyte suspension and equal volume of 25B-NBF (4 μM) in Krebs–Henseleit buffer were mixed into 96-well plates and incubated in triplicate for 0, 5, 15, 30, 60, 120, or 180 min in a CO_2_ incubator at 37 °C. Incubation was quenched by addition of 120 µL ice-cold acetonitrile to each well, and the samples were centrifuged at 15,000× *g* for 10 min at 4 °C after 5 min sonication. Then 100 µL of supernatants were vortex-mixed with equal volume of water and 2 μL aliquots were analyzed by an LC–MS/MS system. Honokiol (2 µM) was separately incubated for the positive control of this system. The peak area ratios of substances versus IS at each sampling point were used in subsequent calculations of parameters. The following equations were used in the calculation of elimination parameters, including *t*_1/2_, *Cl_int_*, *Cl_hep_*, and hepatic extraction ratio of 25B-NBF.

(1) $$t_{\frac{1}{2}}\left(\min\right)=\frac{ln2}{-k\;\left(\mathrm{elimination}\;\mathrm{slope}\right)}$$

(2) $$Cl_{int}\left(\mathrm{mL}/\min/\mathrm{kg}\right)=\frac{ln2}{t_{\frac{1}{2}}}\times \frac{\mathrm{mL}\;\mathrm{incubation}}{\mathrm{hepatocyte}\;\left(10^{6}\;\mathrm{cells}\right)}\times \frac{139\;\times 10^{6}\mathrm{cells}}{g\;\mathrm{liver}}\times \frac{25.7\;g\;\mathrm{liver}}{\mathrm{kg}\;\mathrm{body}\;\mathrm{weight}}$$

(3) $$Cl_{hep}=\frac{Q_{h}\times Cl_{int}}{Q_{h}+Cl_{int}},\;\;Q_{h}=20.7\;\mathrm{mL}/\min/\mathrm{kg}$$

(4) $$\mathrm{hepatic}\;\mathrm{extraction}\;\mathrm{ratio}=\frac{Cl_{hep}}{Q_{h}}$$

*Q_h_* refers to hepatic blood flow [29]. Comparing to the general classification of hepatic extraction ratio, the metabolic rate of 25B-NBF was evaluated [30].

### 4.3. Metabolite Identification in Human Hepatocytes

An amount of 60 µL 25B-NBF (20 μM) in Krebs-Henseleit buffer and human hepatocyte suspensions (60 μL; 1.0 × 10^6^ cells/mL) were mixed in 96-well plates and incubated in triplicate for 2 h in a CO_2_ incubator at 37 °C. Incubation was quenched by addition of 120 µL ice-cold acetonitrile to each well, and then the samples were centrifuged at 15,000× *g* for 10 min at 4 °C after 5 min sonication. Then the supernatants (200 µL) were dried using a speed-vac and residues were reconstituted with 10% methanol (100 µL). 5 μL Aliquots of each sample were analyzed using an LC–HRMS system.

### 4.4. Metabolism of 25B-NBF in Human cDNA-Expressed CYPs and UGTs

25B-NBF was incubated with 12 human cDNA-expressed CYP enzymes (CYPs 1A1, 1A2, 2A6, 2B6, 2C8, 2C9, 2C19, 2D6, 2E1, 2J2, 3A4, and 3A5) to determine CYP enzymes involved in the metabolism of 25B-NBF. The 95 µL of reaction mixtures were prepared as follow: 10 µL of 100 µM 25B-NBF stock solution, 10 µL of CYPs (40 pmol), 4 µL of 250 mM magnesium chloride, and 71 µL of potassium phosphate buffer (pH 7.4, 50 mM). The incubation was started by addition of NADPH-generating system (5 µL), and the mixtures were incubated for 30 min at 37 °C in triplicate.

For the characterization of the UGT enzymes involved in the glucuronidation of 25B-NBF to M9, 25B-NBF was incubated with 12 human cDNA-expressed UGT enzymes (UGTs 1A1, 1A3, 1A4, 1A6, 1A7, 1A8, 1A9, 1A10, 2B4, 2B7, 2B10, and 2B15). The 95 µL of reaction mixtures were prepared as follow: 10 µL of 100 µM 25B-NBF stock solution, 10 µL of UGTs (2.5 mg/mL), 1 µL of 2.5 mg/mL alamethicin, 4 µL of 5 mM magnesium chloride, and 70 µL of Tris-HCl buffer (pH 7.4, 50 mM). The reaction was initiated by addition of 25 mM UDPGA (5 µL), and the mixtures were incubated for 30 min at 37 °C in triplicate. 

By adding 100 µL of ice-cold methanol (containing 100 ng/mL of IS), the reaction was terminated. After centrifugation at 10,000× *g* for 4 min at 4 °C, 150 µL of supernatant was dried under N_2_ gas. The residue was reconstituted in 50 µL of 45% methanol and a 5 µL of aliquot was injected into the LC–HRMS system.

### 4.5. LC–MS Analyses

To assess the metabolic stability of 25B-NBF, we used an Agilent 1290 Infinity UPLC coupled with Agilent 6495 triple quadrupole MS (Agilent Technologies, Santa Clara, CA, USA) in the quantification of 25B-NBF. For the chromatographic separation, 5% methanol containing 0.1% formic acid and 95% methanol containing 0.1% formic acid were used as mobile phase A and mobile phase B, respectively, and a Halo C_18_ column (2.1 × 50 mm, 2.7 μm; Advanced Materials Technology, Wilmington, DE, USA) was used as a stationary phase. The flow rate used was 0.3 mL/min, and gradient elution was performed as follows: 10% B for 0–1 min, 10–90% B for 1–2.5 min, 90% B held for 2.5–4 min, 90–10% B for 4–4.1 min, and 10% B for 4.1–6 min. MS spectra were acquired in positive-ion mode using electrospray ionization (ESI). ESI conditions were optimized as follows: gas temperature 200 °C, gas flow 16 L/min, nebulizer 40 psi, sheath gas temp 380 °C, sheath gas flow 12 L/min, capillary voltage 4.5 kV, and nozzle voltage 500 V. Selective reaction monitoring transitions were 367.9→242.9 at a collision energy (CE) of 20 for 25B-NBF and 531.2→489.2 at a CE of 34 for ketoconazole.

For 25B-NBF metabolites identification and their quantification, we used a Nexera-X2 HPLC system (Shimadzu, Kyoto, Japan) coupled to a Q-Exactive Orbitrap mass spectrometer (Thermo Fisher Scientific Inc., Waltham, MA, USA). The mass spectrometer was calibrated using ready-to-use calibration solution immediately before sample analysis. For the chromatographic separation, a Halo C18 column (2.1 × 100 mm, 2.7 μm; Advanced Materials Technology, Wilmington, DE, USA), 5% methanol in 0.1% formic acid (mobile phase A), and 95% methanol in 0.1% formic acid (mobile phase B) were used. Gradient elution was performed as follows: 10% B for 0–1.5 min, 10–50% B for 1.5–10 min, 50–90% B for 10–12 min, 90% B for 12–15 min, 90–10% B for 15–15.2 min, and 10% B for 15.2–18 min. The flow rate was 0.3 mL/min. Column and autosampler were maintained at 40 °C and 4 °C, respectively.

A heated electrospray ionization (HESI) source was interfaced and the MS spectra were obtained in positive mode. For the metabolite identification, HESI source conditions were optimized as follows: 35 for sheath gas flow rate (arbitrary units), 10 for auxiliary gas flow rate (arbitrary units), 4 kV for spray voltage, and 350 °C for heater temperature. Xcalibur software (Thermo Fisher Scientific Inc.) was used in acquiring and processing MS spectra. Full MS spectra were acquired at a resolution of 70,000 (from *m*/*z* 100 to *m*/*z* 1500), and data-dependent MS/MS spectra were obtained at a resolution of 35,000 (normalized collision energies at 23 and 28). Prediction of fragment ions was aided by Mass Frontier (version 6.0; HighChem Ltd., Bratislava, Slovakia). Each metabolite was identified allowing 5 ppm mass error from the theoretical value. Owing to the absence of authentic standard of 25B-NBF metabolites, the formation rates of each metabolite were calculated using the standard curve of 25B-NBF.

## 5. Conclusions

25B-NBF was extensively metabolized into 33 metabolites by hydroxylation, *O*-demethylation, bis-*O*-demethylation, *N*-dearylation, cysteine conjugation, glucuronidation, sulfation, and acetylation alone or in combination in human hepatocytes. 2C-B, one of the most widespread NPSs, was identified as a major metabolite of 25B-NBF. Multiple CYPs including CYP1A1, CYP1A2, CYP2B6, CYP2C9, CYP2C19, CYP2D6, CYP2J2, and CYP3A4 were involved in hydroxylation, *O*-demethylation, bis-*O*-demethylation, and *N*-dearylation of 25B-NBF. UGT2B7 played a prominent role in glucuronidation of 25B-NBF to M9. The present study shows that it is necessary to determine the metabolites as well as 25B-NBF in biological samples to detect 25B-NBF abuse. These results may help to predict pharmacokinetic profiles aof 25B-NBF in humans and to address which metabolites should be synthesized for the analysis of 25B-NBF and metabolites in biological samples.

## Figures and Tables

**Table 1:** Retention times, molecular formulae, theoretical molecular ion masses, mass accuracies, fragment ions, and biotransformations of 25B-NBF [2-(4-bromo-2,5-dimethoxyphenyl)-*N*-(2-fluorobenzyl)ethanamine]metabolites.

ID	RT (min)	Formula	Exact Mass ([M + H]^+^)	Error (ppm)	Fragment Ions	Biotransformation
25B-NBF	9.9	C_17_H_19_BrFNO_2_	368.0656	−0.5	243.0014, 227.9780, 109.0451	Parent
M1	8.8	C_17_H_19_BrFNO_3_	384.0605	1.0	260.0281, 243.0015, 227.9780, 125.0399	Monohydroxylation
M2	9.2	C_17_H_19_BrFNO_3_	384.0605	−0.3	260.0281, 243.0016, 227.9780, 125.0399	Monohydroxylation
M3	9.4	C_17_H_19_BrFNO_3_	384.0605	0.0	260.0280, 243.0015, 227.9780, 125.0399	Monohydroxylation
M4	12.7	C_17_H_19_BrFNO_3_	384.0605	−0.8	260.0280, 243.0014, 227.9780, 125.0398	Monohydroxylation
M5	8.1	C_16_H_17_BrFNO_2_	354.0499	-0.3	228.9858, 213.9626, 109.0451	*O*-Demethylation
M6	8.8	C_16_H_17_BrFNO_2_	354.0499	0.3	228.9859, 213.9624, 109.0451	*O*-Demethylation
M7	6.3	C_15_H_15_BrFNO_2_	340.0343	−0.9	214.9703, 109.0451	Bis-*O*-demethylation
M8	6.1	C_10_H_14_BrNO_2_	260.0281	-0.8	243.0014, 227.9780	*N*-Debenzylation
M9	11.8	C_23_H_27_BrFNO_8_	544.0977	−1.8	368.0292	Glucuronidation
M10	8.4	C_20_H_24_BrFN_2_O_4_S	487.0697	0.4	243.0015, 227.9780	Cystein conjugation
M11	7.3	C_16_H_17_BrFNO_3_	370.0449	−1.4	245.0046, 228.9859, 125.0399	Monohydroxylation + *O*-Demethylation
M12	7.7	C_16_H_17_BrFNO_3_	370.0449	−0.8	245.0046, 228.9859, 125.0398	Monohydroxylation + *O*-Demethylation
M13	8.0	C_16_H_17_BrFNO_3_	370.0449	−0.3	245.0046, 228.9858, 125.0399	Monohydroxylation + *O*-Demethylation
M14	8.3	C_16_H_17_BrFNO_3_	370.0449	−0.5	245.0046, 228.9858, 125.0398	Monohydroxylation + *O*-Demethylation
M15	8.6	C_16_H_17_BrFNO_3_	370.0449	−0.8	245.0046, 228.9859, 125.0399	Monohydroxylation + *O*-Demethylation
M16	11.5	C_16_H_17_BrFNO_3_	370.0449	−0.8	228.9859, 214.9702, 109.0451	Monohydroxylation + *O*-Demethylation
M17	11.8	C_16_H_17_BrFNO_3_	370.0449	0.0	245.0046, 228.9858, 125.0399	Monohydroxylation + *O*-Demethylation
M18	7.6	C_23_H_27_BrFNO_9_	560.0926	0.7	384.0605, 243.0013, 125.0398	Monohydroxylation + Glucuronidation
M19	7.9	C_23_H_27_BrFNO_9_	560.0926	0.4	384.0606, 260.0280, 125.0399	Monohydroxylation + Glucuronidation
M20	13.0	C_23_H_27_BrFNO_9_	560.0926	0.4	384.0601, 243.0013, 125.0399	Monohydroxylation + Glucuronidation
M21	6.3	C_22_H_25_BrFNO_9_	546.0769	0.4	370.0447, 352.0345, 228.9859, 109.0450	Monohydroxylation + *O*-Demethylation + Glucuronidation
M22	6.5	C_22_H_25_BrFNO_9_	546.0769	0.4	370.0447, 246.0124, 228.9861, 125.0399	Monohydroxylation + *O*-Demethylation + Glucuronidation
M23	11.0	C_22_H_25_BrFNO_9_	546.0769	1.1	370.0451, 228.9859, 214.9702, 109.0450	Monohydroxylation + *O*-Demethylation + Glucuronidation
M24	8.3	C_16_H_17_BrFNO_6_S	450.0017	0.9	370.0449, 228.9857, 125.0398	Monohydroxylation + *O*-Demethylation + Sulfation
M25	8.7	C_16_H_17_BrFNO_6_S	450.0017	0.2	370.0449, 228.9857, 125.0399	Monohydroxylation + *O*-Demethylation + Sulfation
M26	11.4	C_16_H_17_BrFNO_6_S	450.0017	−0.7	370.0439, 228.9857, 109.0451	Monohydroxylation + *O*-Demethylation + Sulfation
M27	3.2	C_9_H_12_BrNO_2_	246.0124	−1.2	228.9859	*O*-Demethylation + *N*-Debenzylation
M28	3.8	C_9_H_12_BrNO_2_	246.0124	0.4	228.9858	*O*-Demethylation + *N*-Debenzylation
M29	7.4	C_22_H_25_BrFNO_8_	530.0820	0.6	354.0498, 228.9859	*O*-Demethylation + Glucuronidation
M30	8.8	C_16_H_17_BrFNO_5_S	434.0068	−0.2	354.0499, 308.9427, 228.9859, 109.0451	*O*-Demethylation + Sulfation
M31	9.6	C_16_H_17_BrFNO_5_S	434.0068	−0.2	354.0501, 308.9427, 228.9859, 109.0451	*O*-Demethylation + Sulfation
M32	4.1	C_21_H_23_BrFNO_8_	516.0664	0.2	340.0343, 214.9701, 109.0450	Bis-*O*-demethylation + Glucuronidation
M33	11.7	C_12_H_16_BrNO_3_	302.0386	−0.3	260.0280, 243.0015, 227.9781	*N*-Dearylation + Acetylation

## Supplementary Materials

The following are available online. Figure S1, MS/MS spectra of metabolites of 25B-NBF identified after incubation with human hepatocytes for 2 h at 37 °C in a CO_2_ incubator. (**A**) cysteine conjugate of 25B-NBF (M10). (**B**) hydroxy-*O*-demethyl-25B-NBF (M11–15 and M17); (**C**) hydroxy-*O*-demethyl-25B-NBF (M16). Figure S2, MS/MS spectra of metabolites of 25B-NBF identified in human hepatocytes after incubation for 2 h at 37 °C in a CO_2_ incubator. (**A**) hydroxy-M9 (M18–M20); (**B**) hydroxy-*O*-demethyl-M9 (M21 and M23); (**C**) hydroxyl-O-demethyl-M9 (M22). Figure S3, MS/MS spectra of metabolites of 25B-NBF identified in human hepatocytes after incubation for 2 h at 37 °C in a CO_2_ incubator. (**A**) hydroxy-*O*-demethyl-25B-NBF sulfates (M24 and M25); (**B**) hydroxy-*O*-demethyl-25B-NBF sulfate (M26); (**C**) *O*-demethyl-M8 (M27 and M28). Figure S4, MS/MS spectra of metabolites of 25B-NBF identified in human hepatocytes after incubation for 2 h at 37 °C in a CO_2_ incubator. (**A**) *O*-demethyl-M9 (M29); (**B**) *O*-demethyl-25B-NBF sulfates (M30 and M31); (**C**) M7 glucuronide (M32); (**D**) acetyl-M8 (M33).
