# Supplementary material for: In Vitro Metabolism of 25B-NBF, 2-(4-Bromo-2,5-Dimethoxyphenyl)-N-(2-Fluorobenzyl)ethanamine, in Human Hepatocytes Using Liquid Chromatography–Mass Spectrometry
Source: Molecules. 2019 Feb 25;24(4):818. doi: 10.3390/molecules24040818 (PMC6412758; doi:10.3390/molecules24040818)
Supplement: Supplementary file 1 [file molecules-24-00818-s001.zip › molecules-446228-supplementary.pptx]

## Slide 1
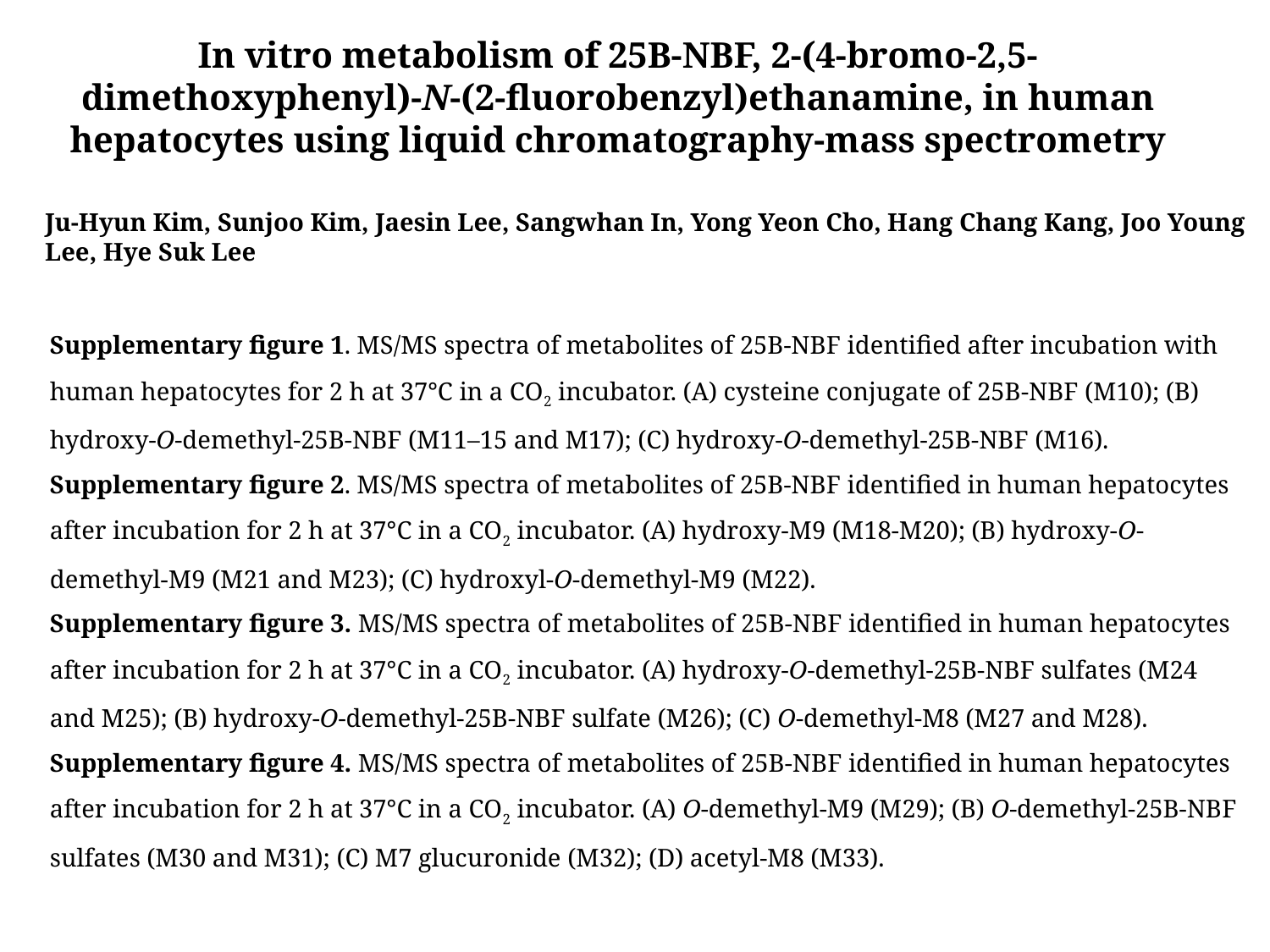

# In vitro metabolism of 25B-NBF, 2-(4-bromo-2,5-dimethoxyphenyl)-N-(2-fluorobenzyl)ethanamine, in human hepatocytes using liquid chromatography-mass spectrometry
Ju-Hyun Kim, Sunjoo Kim, Jaesin Lee, Sangwhan In, Yong Yeon Cho, Hang Chang Kang, Joo Young Lee, Hye Suk Lee
Supplementary figure 1. MS/MS spectra of metabolites of 25B-NBF identified after incubation with human hepatocytes for 2 h at 37°C in a CO2 incubator. (A) cysteine conjugate of 25B-NBF (M10); (B) hydroxy-O-demethyl-25B-NBF (M11–15 and M17); (C) hydroxy-O-demethyl-25B-NBF (M16).
Supplementary figure 2. MS/MS spectra of metabolites of 25B-NBF identified in human hepatocytes after incubation for 2 h at 37°C in a CO2 incubator. (A) hydroxy-M9 (M18-M20); (B) hydroxy-O-demethyl-M9 (M21 and M23); (C) hydroxyl-O-demethyl-M9 (M22).
Supplementary figure 3. MS/MS spectra of metabolites of 25B-NBF identified in human hepatocytes after incubation for 2 h at 37°C in a CO2 incubator. (A) hydroxy-O-demethyl-25B-NBF sulfates (M24 and M25); (B) hydroxy-O-demethyl-25B-NBF sulfate (M26); (C) O-demethyl-M8 (M27 and M28).
Supplementary figure 4. MS/MS spectra of metabolites of 25B-NBF identified in human hepatocytes after incubation for 2 h at 37°C in a CO2 incubator. (A) O-demethyl-M9 (M29); (B) O-demethyl-25B-NBF sulfates (M30 and M31); (C) M7 glucuronide (M32); (D) acetyl-M8 (M33).

## Slide 2
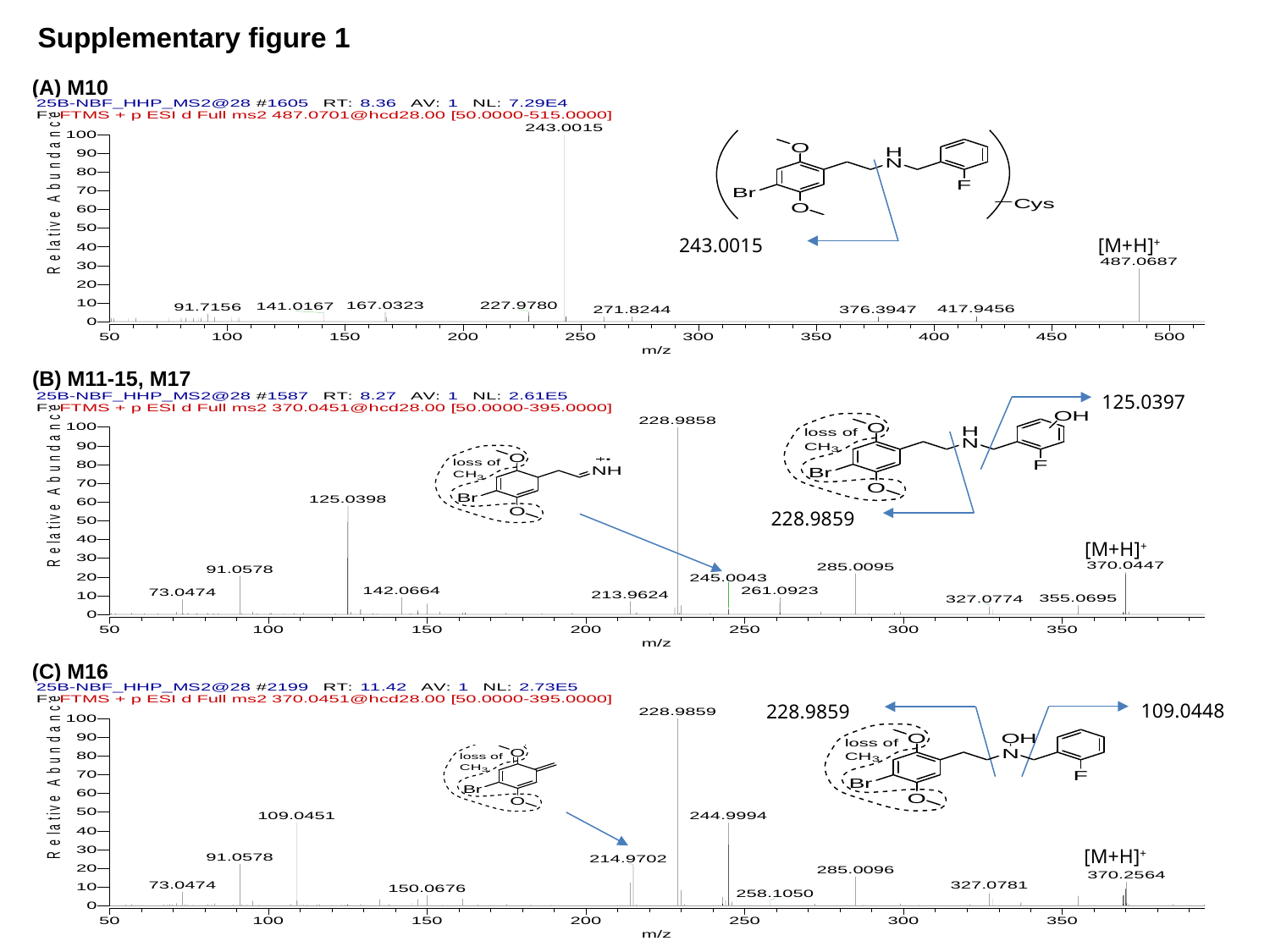

Supplementary figure 1
(A) M10
[M+H]+
243.0015
(B) M11-15, M17
125.0397
228.9859
[M+H]+
(C) M16
109.0448
228.9859
[M+H]+

## Slide 3
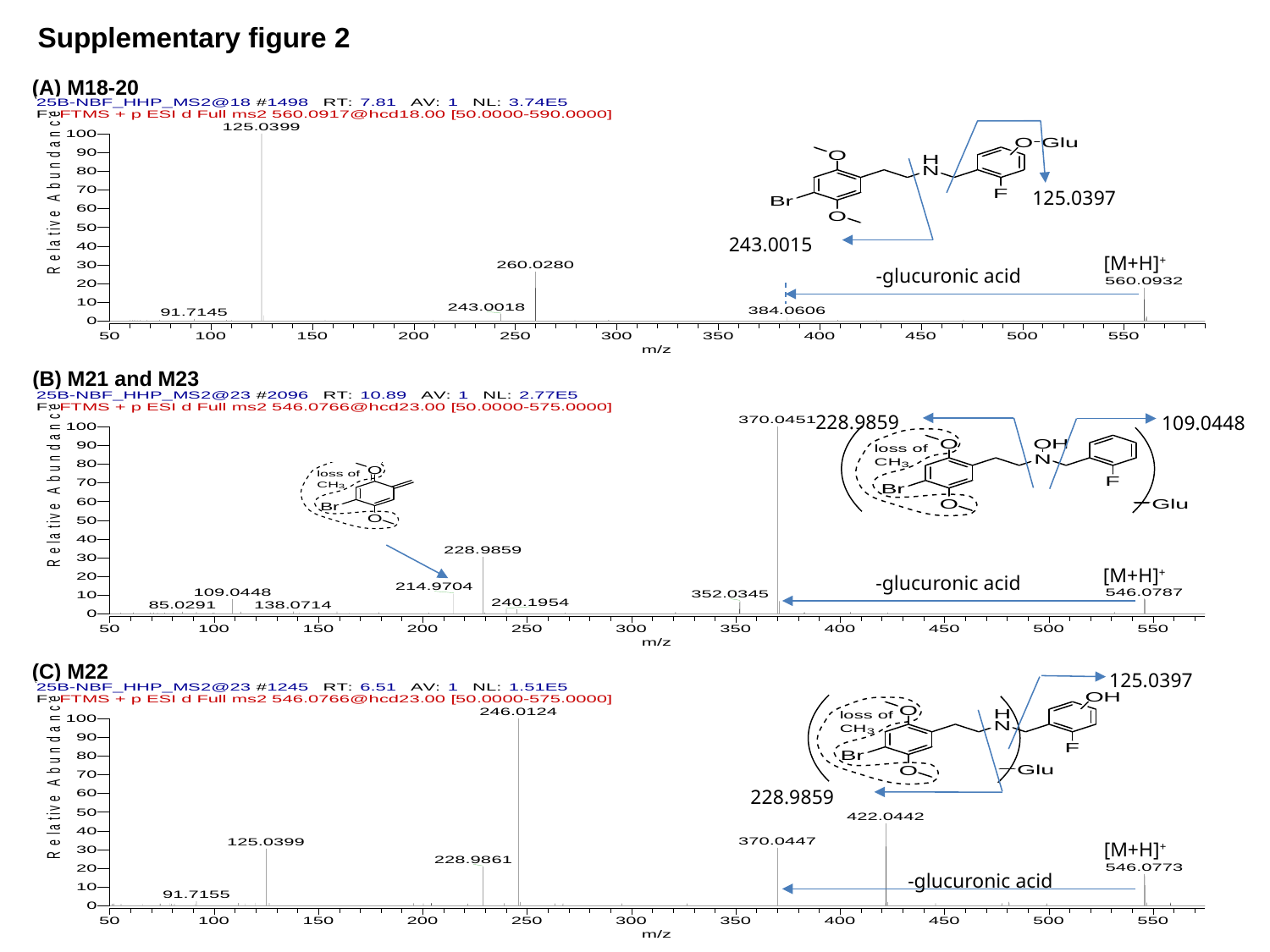

Supplementary figure 2
(A) M18-20
125.0397
243.0015
[M+H]+
-glucuronic acid
(B) M21 and M23
228.9859
109.0448
[M+H]+
-glucuronic acid
(C) M22
125.0397
228.9859
[M+H]+
-glucuronic acid

## Slide 4
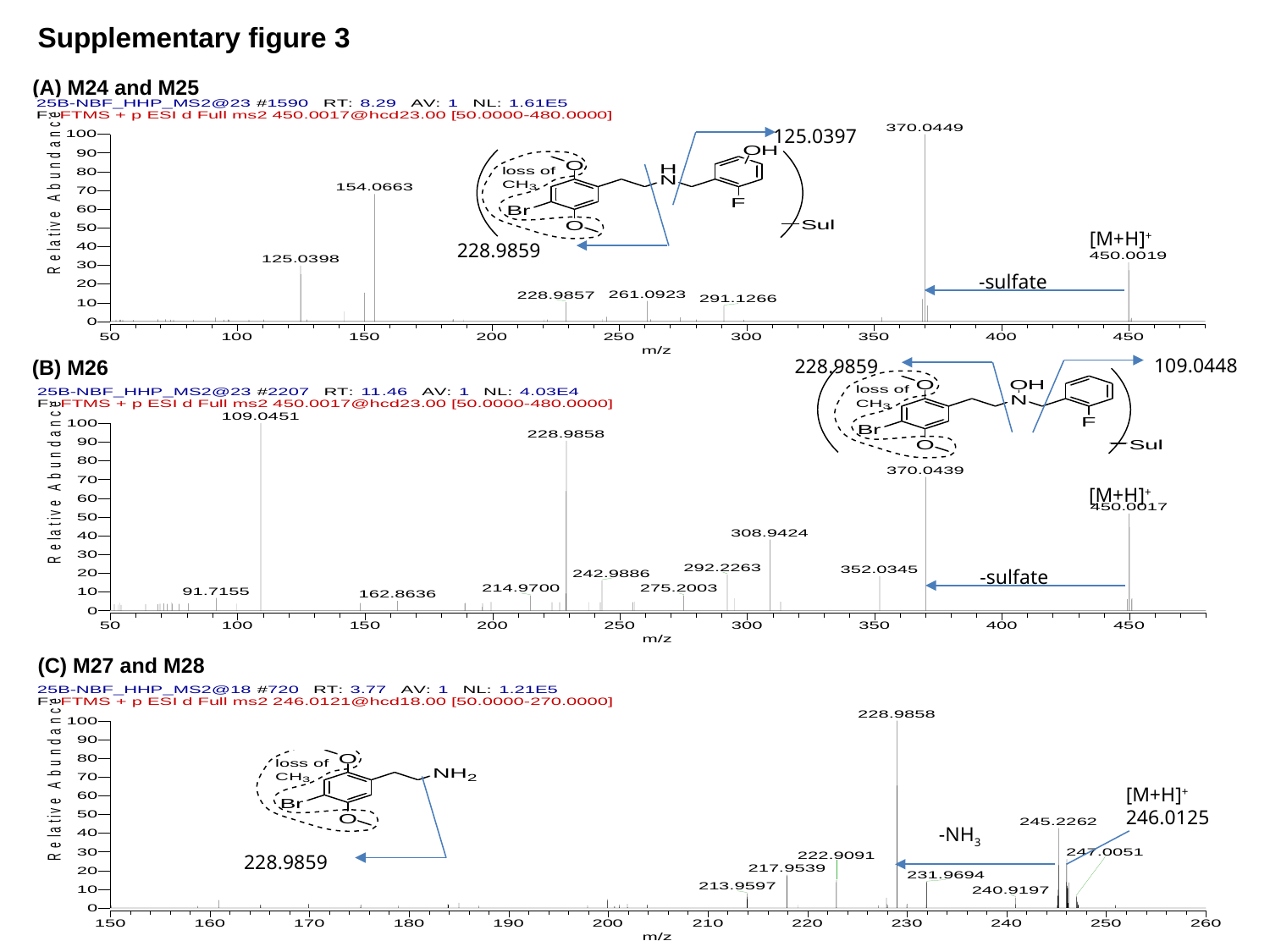

Supplementary figure 3
(A) M24 and M25
125.0397
[M+H]+
228.9859
-sulfate
109.0448
(B) M26
228.9859
[M+H]+
-sulfate
(C) M27 and M28
[M+H]+
246.0125
-NH3
228.9859

## Slide 5
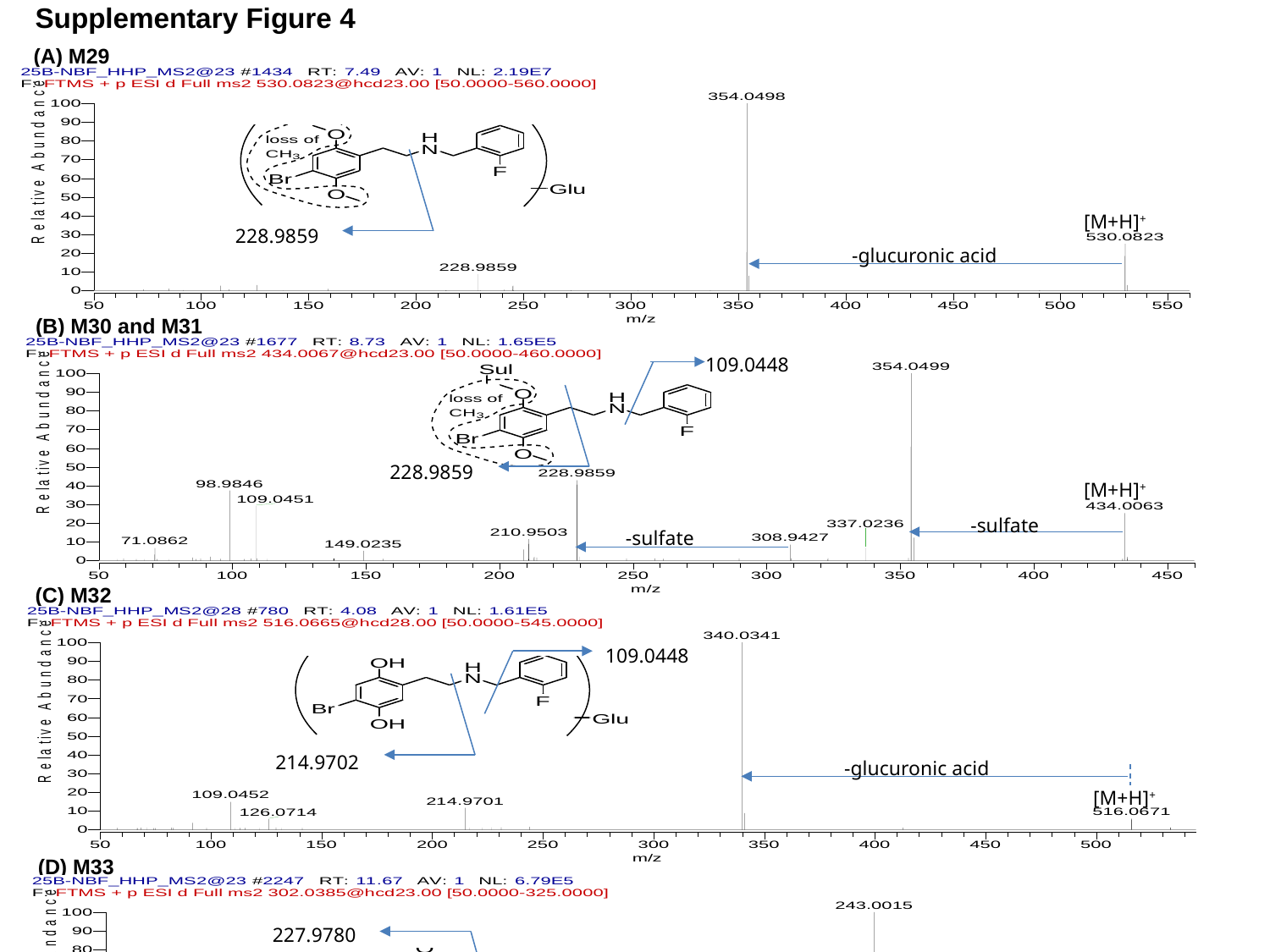

Supplementary Figure 4
(A) M29
[M+H]+
228.9859
-glucuronic acid
(B) M30 and M31
109.0448
228.9859
[M+H]+
-sulfate
-sulfate
(C) M32
109.0448
214.9702
-glucuronic acid
[M+H]+
(D) M33
227.9780
243.0015
[M+H]+
-NH3
-acetyl
